# Transcriptional regulatory network differences between six industrial *Escherichia coli* strains

**DOI:** 10.1016/j.mec.2026.e00283

**Published:** 2026-06-09

**Authors:** Yuan Yuan, Gaoyuan Li, Ying Hefner, Richard Szubin, Jaemin Sung, Irina Rodionova, Bernhard O. Palsson

**Affiliations:** a Shu Chien-Gene Lay Department of Bioengineering, University of California San Diego, La Jolla, CA, 92093, USA; b Bioinformatics and Systems Biology Program, University of California, San Diego, La Jolla, USA; c Department of Pediatrics, University of California, San Diego, La Jolla, CA, USA; d Novo Nordisk Foundation Center for Biosustainability, Technical University of Denmark, Kemitorvet, Building 220, 2800 Kongens, Lyngby, Denmark

## Abstract

*Escherichia coli* strains are widely used across numerous industrial and biotechnological applications. Yet their performance varies substantially in ways that can not be anticipated from genome annotation. Because transcriptional regulatory networks (TRNs) govern cellular functions such as motility, stress responses, metabolic flexibility, and production efficiency, differences in TRN organization and use may underlie many observed phenotypic differences. To investigate TRN differences between strains, we generated a compendium of 433 matched RNA-Seq profiles for six commonly used industrial *E. coli* strains (BL21, C, Crooks, MG1655, W, and W3110) and applied iModulon analysis to compare the state of their TRNs under similar growth conditions. This analysis revealed that core regulatory programs with similar functions are wired differently across the strains, and that the strains engage these programs in distinct ways when exposed to the same environmental challenges. Together, these findings highlight transcriptional regulation diversity underlying phenotypic expression among industrial *E. coli* strains. By providing an integrated view of TRN differences across widely used hosts, this work offers a fundamental basis for interpreting strain-specific behaviors and supports more informed approaches to strain selection and optimization.

## Highlights

• Transcriptome-wide comparison reveals regulatory differences across closely related *E. coli* strains
• Core regulatory programs are conserved but exhibit strain-specific gene composition and wiring
• Strains differentially engage shared regulatory modules under matched environmental conditions
• Transcriptional responses explain phenotypic divergence not captured by genome similarity alone
• Multi-view ICA provides a unified framework for comparing transcriptional regulatory networks across strains

## 1 Introduction

*Escherichia coli* has played a central role in industrial biotechnology, utilized broadly for recombinant protein production, metabolic engineering, and the synthesis of high-value chemicals (Adamczyk and Reed, 2017; Blount, 2015; Jang et al., 2025; Swartz, 2001). Over time, multiple laboratory-adapted and production strains have become established as standard hosts (such as K-12 derivatives and B strains), each shaped by distinct evolutionary histories (Archer et al., 2011; Hayashi et al., 2006; Jeong et al., 2015; Kim et al., 2017; Kuhnert et al., 1995; Lynch et al., 2022). These strains differ in traits such as stress tolerance, metabolic efficiency, and production performance. For example, strain BL21 is widely favored for high-level protein production, whereas K-12 derivatives display differences in motility, acetate formation and other metabolic trains (Archer et al., 2011; Arifin et al., 2014a, Arifin et al., 2014b; Chae et al., 2010; Marisch et al., 2013; Vijayendran et al., 2007; Yoon et al., 2012). Therefore, host strain choice can influence engineering outcomes (Archer et al., 2011; Arifin et al., 2014a; Chae et al., 2010; Marisch et al., 2013; Vijayendran et al., 2007; Yoon et al., 2012). Despite these differences, strain selection often relies on empirical precedent rather than systematic principles because the mechanistic bases for these phenotypic differences are not fully understood.

Previous work has compared industrial *E. coli* strains using genomic analyses, metabolic profiling, and physiological characterization (Archer et al., 2011; Galardini et al., 2017; Marisch et al., 2013; Monk et al., 2016; Sabarly et al., 2011). Collectively, these efforts have shown that strain choice strongly influences production performance and that individual strains possess distinct advantages and limitations (Lee et al., 2020; Marisch et al., 2013; Monk et al., 2016; Yoon et al., 2009). However, most existing knowledge stems from studies focused on specific strains, conditions, or design goals, resulting in a fragmented landscape rather than a unified framework for systematic host selection. Identifying the most suitable strain thus often remains empirical or reliant on broad heuristics (Lee et al., 2007, 2020). Furthermore, different biological layers have been profiled to varying extents: while genomic and metabolic characterization is comparatively well developed, other layers remain less explored, leaving gaps in how these scales relate to one another (Covert et al., 2008; Monk et al., 2016; Orth et al., 2010; Rasko et al., 2008).

Among these, transcriptome-level comparisons have been especially limited, yet they offer direct insight into how strains activate and coordinate their genetic potential in response to specific environments. Transcriptomic analysis reveals regulatory features, such as differences in regulatory wiring, co-regulated gene sets, and the magnitude or timing of pathway activation, that cannot be inferred from genome content alone. These distinctions arise even when strains share highly similar genetic repertoires, and they can potentially determine strain-specific physiological behavior by influencing how similar genetic potential is deployed, shaping how cells allocate resources and balance metabolic or stress demands. While genome-scale metabolic models and comparative genomics have been instrumental in predicting metabolic capabilities, these approaches typically define the metabolic 'possibility space' based on gene presence. They assume fixed regulatory structures and do not account for dynamic transcriptional control and regulatory logic that governs resource allocation. As a result, they may not fully explain phenotypic divergence among closely related strains that share highly similar gene content. Incorporating systematic comparisons of transcriptional regulatory networks (TRNs) across industrial strains therefore complements existing genomic and phenotypic observations, providing a more coherent and mechanistic basis for interpreting their diverse behaviors and guiding strain selection.

Advances in high-throughput RNA sequencing have enabled the generation of large transcriptomic datasets that can be used to characterize bacterial TRNs at scale. Independent Component Analysis (ICA) has emerged as a powerful method for extracting regulatory structure from such datasets (Rychel et al., 2020; Sastry et al., 2019; Yuan et al., 2024). Comparative benchmarking of transcriptional regulatory network inference methods has shown that ICA-based methods perform strongly in recovering known gene modules (Saelens et al., 2018). Subsequent studies have further demonstrated that ICA-derived modules are robust across independent datasets (Cantini et al., 2019; Zhang et al., 2005). ICA has also been applied across diverse organisms, including yeast, human cancer systems, and multiple bacterial species, supporting its generalizability and biological interpretability (Catoiu et al., 2025; Choe et al., 2024; Engreitz et al., 2010; Karczewski et al., 2014; Kong et al., 2008; Sompairac et al., 2019). ICA decomposes the gene expression matrix **X** into two matrices: the iModulon matrix **M** and the activity matrix **A**. iModulons, extracted from the **M** matrix, are groups of genes with coordinated expression patterns and capture the organization of regulatory programs. Their activity profiles in the **A** matrix reflect the collective regulatory behavior of these genes across conditions and how the TRN responds to environmental or physiological cues. This framework provides a top–down representation of both the organization and activity of the TRN, offering a holistic view of transcriptional regulation (Catoiu et al., 2025; Lamoureux et al., 2023; Sastry et al., 2024).

In this study, we generated a matched RNA-Seq compendium of 433 profiles for six representative and commonly used *E. coli* strains: BL21 (Jeong et al., 2009, 2015; Studier et al., 2009), C (Jantama et al., 2008a, 2008b; Monk et al., 2016), Crooks (Duque-Sarango et al., 2023; El-Shanshoury et al., 2011; Gędas et al., 2024; How et al., 2013), MG1655, W (Archer et al., 2011; Arifin et al., 2014b) and W3110 (Dong et al., 2017; Hayashi et al., 2006; Vijayendran et al., 2007; Vo and Park, 2022), profiling each under the same diverse set of environmental and stress conditions. We applied Multi-View ICA to jointly decompose these datasets and identify iModulons that are conserved across strains as well as those that are strain specific (Richard et al., 2020). By examining both iModulon gene membership and condition-dependent activity, we characterize how core regulatory programs differ among strains and how each strain engages its regulatory network under shared stimuli. This work establishes a systematic transcriptomic foundation for understanding regulatory diversity in industrial *E. coli* and for interpreting strain-specific phenotypes in the context of their underlying TRNs.

## 2 Results

### 2.1 Exploring genomic similarities and substrate-utilization variation among strains

**Fig. 1:**
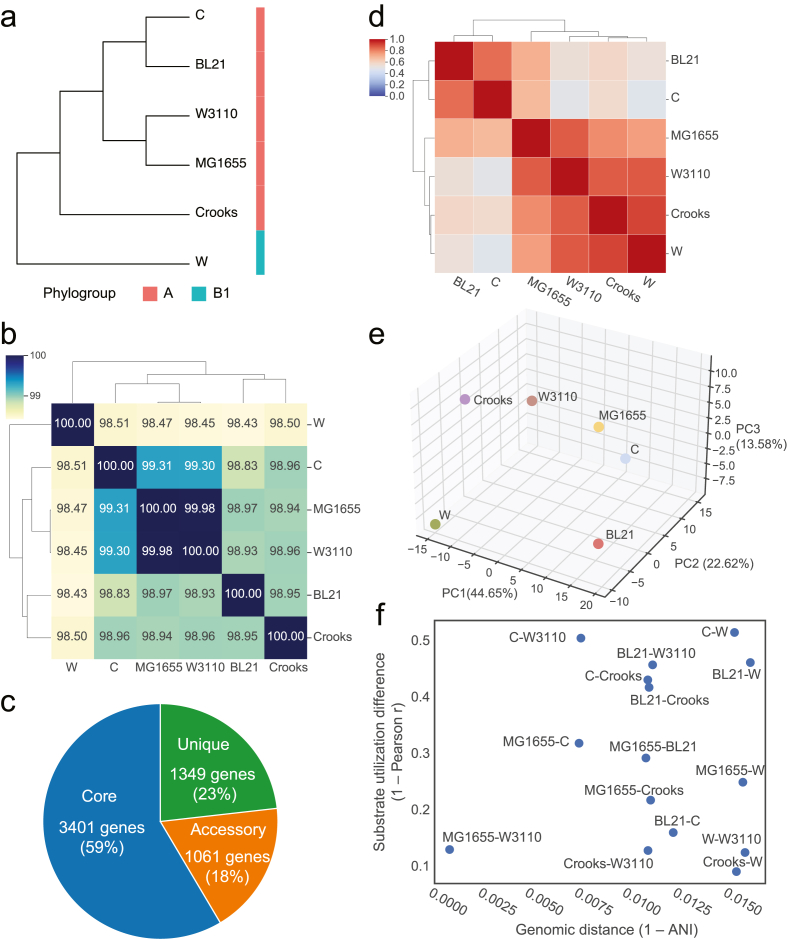
Genomic Relationships and Substrate Utilization Differences of the Six Industrial *E. coli Strains. a) Phylogenetic tree based on whole-genome SNP phylogeny of the six E. coli strains included in this study. Five of the strains belong to phylogroup A, while strain W belongs to phylogroup B1.****b)****Average nucleotide identity (ANI) clustermap showing pairwise genomic similarity between the strains.****c)****Pie chart showing the pangenome composition of the six strains. Number of genes and fraction of the core (shared across all strains), accessory (shared by 2-5 strains) and unique genes are indicated.****d)****Clustermap showing substrate utilization similarity (Pearson r) of the six strains from growth information in Biolog data, plates PM1, PM2A, PM3B and PM4A.****e)****Three dimensional PCA plot of Biolog substrate utilization profiles.****f)****Comparison of genomic distance (defined as 1 - ANI) and Biolog-based substrate utilization difference (defined as 1 - Pearson r between normalized AUC values) between strain pairs indicated as “Strain1-Strain2” on the plot.*

The six *E. coli* strains examined here have been widely used in industrial and laboratory applications. All strains have complete genome sequences available (Archer et al., 2011; Clark et al., 2016; GenBank accession U00096.3; Freddolino et al., n.d.; Hayashi et al., 2006; Jeong et al., 2009; Król et al., 2019). To establish their genomic relatedness, we first constructed a whole-genome SNP phylogeny (Fig. 1a). MG1655 and W3110 fall within the same clade, BL21 groups closely with strain C, and strain W, the only representative of phylogroup B1, forms the most distinct lineage (Archer et al., 2011). We then quantified nucleotide-level similarity using pairwise average nucleotide identity (ANI) (Konstantinidis and Tiedje, 2005) (Fig. 1b). MG1655 and W3110 shared >99.9% ANI, and all strain pairs exceeded 98%, confirming that these strains are highly similar at the sequence level. To assess how much gene-content variation exists within this narrow ANI range, we constructed a pangenome using CD-HIT (Fu et al., 2012) (Fig. 1c). Across the six strains, we identified 5811 non-redundant coding sequences, of which 59% were present in all genomes. The remaining genes were distributed between accessory (present in 2–5 strains) and strain-specific categories. Functional COG distributions for core, accessory, and strain-specific genes are shown in Fig. S1.

Despite their highly similar genome sequences, these strains differ markedly in their industrial performance (Archer et al., 2011; Han et al., 2012; Kim et al., 2017; Monk et al., 2016; Yoon et al., 2012). To quantify phenotypic differences, we profiled substrate utilization ​across diverse carbon, nitrogen, phosphorus, and sulfur sources using phenotypic microarray plates PM1, PM2A, PM3B, and PM4A from Biolog (Bochner et al., 2001; Shea et al., 2012). Pairwise correlations of normalized AUC values (Fig. 1d) showed that some strains share highly similar utilization profiles (e.g., BL21 and C), whereas others diverge substantially. A PCA of the Biolog data (Fig. 1e) further revealed that each strain occupies a distinct region of PCA space, indicating substantial variation in substrate-dependent metabolic activity. These differences also varied by nutrient class. MG1655 and W3110 exhibited highly similar carbon-utilization patterns, whereas BL21 and C were most similar in their utilization of nitrogen and sulfur compounds (Fig. S2).

To relate these phenotypic differences to genomic similarity, we compared genomic distance (1 – ANI) with differences in substrate-utilization profiles (1 – Pearson correlation) for each strain pair (Fig. 1f). MG1655 and W3110 clustered near the origin, reflecting their nearly identical genome sequences and highly similar substrate-use behaviors. However, many other strain pairs displayed strong differences between genomic distances and substrate utilization similarities. For example, Crooks and W are more distant in nucleotide identity yet display similar substrate-use profiles, whereas strain C is genomically close to MG1655/W3110 but differs markedly in substrate utilization. Similar mismatches occurred within individual nutrient classes (Fig. S3).

These results demonstrate that the six strains exhibit substantial variation in substrate-utilization behavior despite their highly similar genome sequences. This disconnect is consistent with their known differences in growth, productivity, and stress tolerance in practical settings. These observations underscore that genome-level information alone is insufficient to understand how closely related strains behave physiologically, motivating the need for transcriptome-level analyses to uncover regulatory and expression-based drivers of these phenotypic differences.

### 2.2 Mapping transcriptional regulatory architecture across industrial strains using transcriptomic analysis

Given the substantial phenotypic differences observed between these closely related strains, we examined transcriptome-wide expression patterns, as regulatory and expression differences may provide mechanistic explanations not evident from the genome sequences alone. Whereas the genome defines each strain's genetic potential, the transcriptome reflects how that potential is deployed under specific environmental conditions. Variation at the transcriptome level can propagate into downstream differences in metabolism, stress tolerance, and growth behavior.

**Fig. 2:**
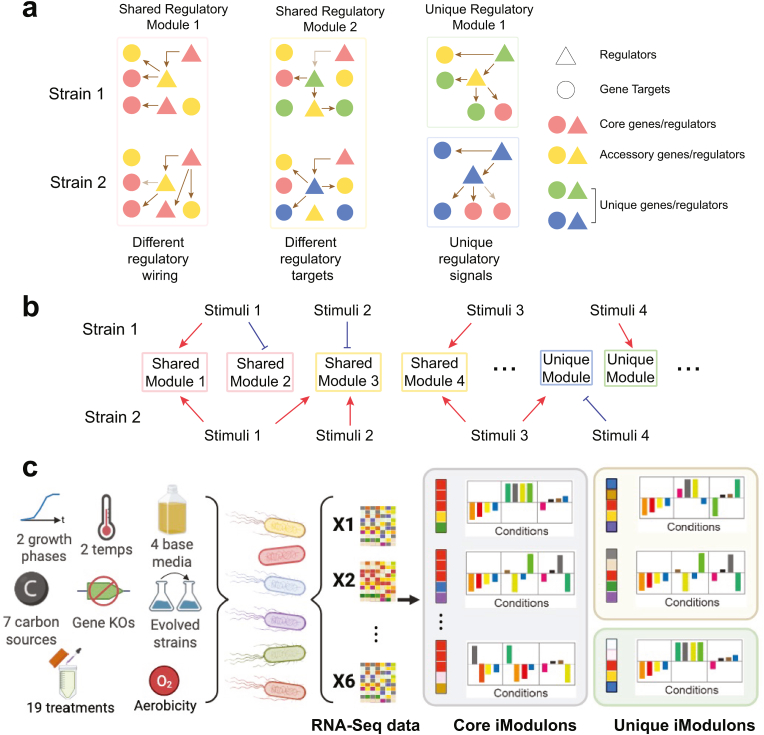
Overview of Transcriptional Regulatory Differences across Strains and iModulon Analysis. a) Schematic illustrating the different ways transcriptional regulatory networks can differ across strains. Regulatory programs may be rewired even with similar gene targets or include different gene targets. There can also be strain-specific modules arising from unique genes or pathways. **b)** Diagram showing how regulatory modules can exhibit distinct activation patterns across strains under the same experimental conditions. **c)** Overview of the experimental conditions, and the Multi-View ICA-based workflow used to extract regulatory modules (iModulons). A diverse set of experimental conditions was used to generate matched RNA-Seq profiles for all six strains. After quality control, the transcriptomes from each strain were jointly decomposed using Multi-View ICA (details described in Methods). We identified 15 core iModulons present across all strains as well as strain-specific iModulons unique to individual strains.

Transcriptomic variation can arise even when gene repertoires are highly similar (Dugar et al., 2013; Hazen et al., 2017; Kopf et al., 2015; Monk et al., 2016; Wurtzel et al., 2012). Strains may encode the same regulators but connect them to different target genes (regulatory wiring differences), or they may activate regulatory programs to different extents under identical conditions (transcription activation differences) (Cummings et al., 2006; Gao et al., 2023; González-Torres et al., 2015; Hazen et al., 2017). Strain-specific regulatory elements may also alter how environmental cues are sensed or interpreted (Fig. 2a and b). As a result, strains with nearly identical genomes can organize and engage their regulatory networks differently, leading to distinct gene-expression responses under the same perturbations. These differences are largely unexplored for these industrial strains. This lack of information motivates a systematic, transcriptome-level comparison of regulatory structure and its use across strains.

To support this analysis, we generated RNA-seq datasets designed to engage broad and diverse transcriptional responses. We profiled each strain across a matched set of conditions varying in stressors, carbon sources, media formulations, and nutrient supplements, and incorporated previously published datasets containing gene knockouts and evolved variants (Gao et al., 2023; Kavvas et al., n.d.; Monk et al., 2016) (Fig. 2c). Across all sources, this approach yielded a large, condition-matched compendium of 433 transcriptomes. This dataset spans a wide range of conditions that do not directly align with those represented by the phenotypic assays described above, but instead enable systematic characterization of regulatory programs across varied contexts. After quality control, each strain retained 69–76 high-quality RNA-seq profiles, with comparable coverage of conditions across strains. We then applied ICA to decompose the transcriptomes into iModulons. An iModulon is a group of genes that are co-regulated across conditions and collectively represent a biologically meaningful transcriptional program within the TRN (Lamoureux et al., 2023; Sastry et al., 2019, 2024). To jointly analyze all six strains, we used a Multi-View ICA framework (Methods), which identifies components consistently shared across datasets while preserving strain-specific signals. The decomposition is performed directly on the RNA-seq data without imposing curated regulatory networks as constraints. Gene annotations were assigned using eggNOG, and for strain MG1655, known regulatory interactions from RegulonDB were available and were used for post hoc interpretation of iModulon gene membership. Thus, regulatory structure emerges in a data-driven manner, enabling systematic comparison across strains, including those lacking curated TRN maps. This approach yielded 15 core iModulons, which are iModulons that are present in all strains, along with a small number of strain-specific iModulons present only in select strains that captured unique regulatory signals.

These iModulons provided a unified framework for mapping the TRNs of all six strains. Because each iModulon represents a coherent regulatory program, they allow direct comparison of how cellular functions are activated and which genes contribute to those functions across strains. By examining both the iModulon gene memberships and condition-dependent activities, we characterized differences in regulatory structure and in the deployment of regulatory programs across strains. In the sections that follow, we focus on examining core iModulon memberships and activities to characterize how TRN organization and regulatory engagement vary across strains, offering transcriptome-level insights that may underlie phenotypic differences not evident from genomic analyses alone. The data set and data analytics provided will enable further studies based on specific questions posed about strain differences.

### 2.3 Strain-specific TRN structure revealed by differential core iModulon gene memberships

**Fig. 3:**
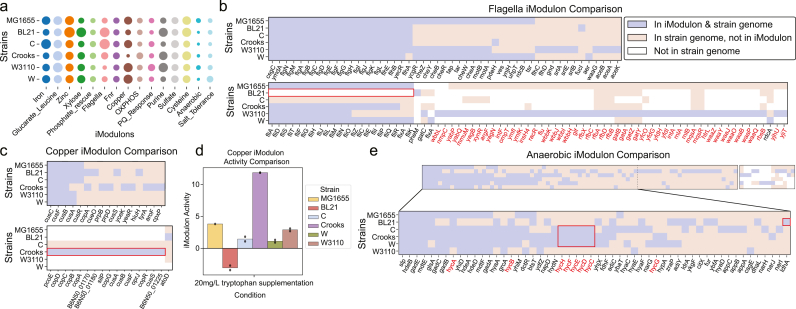
iModulon Composition Differences Across Strains a) *Bubble matrix showing the conservation level of all core iModulons across the six industrial E. coli strains. Rows represent strains and columns represent specific iModulons. Bubble size corresponds to conservation score of the iModulon in a strain. This score is defined as the fraction of genes in that iModulon that are conserved across all strains (# of genes shared in the iModulons of all strains/# of genes in the iModulon). Bubble colors are used to distinguish between iModulons to increase contrast but have no biological meaning.****b)****Comparison of the Flagella iModulon gene membership across strains. Genes discussed in more detail in the main text are highlighted in red.****c)****Comparison of the Copper iModulon membership across strains. Genes discussed in more detail in the main text are highlighted in red.****d)****Activity of the Copper iModulons across all strains under the tryptophan supplementation condition (*20 mg/L *tryptophan supplementation).****e)****Comparison of the Anaerobic iModulon membership across strains. The full composition plot is provided in*Fig. S4*. Part of the figure with the genes discussed in the Results section are enlarged and genes discussed in detail are highlighted in red.*

Although these six industrial strains show extensive genomic similarity (Fig. 1), their iModulon gene memberships revealed clear differences in the structure of their TRNs (Fig. 3). Because each iModulon represents a co-regulated gene set inferred from transcriptomic structure, comparing its membership across strains provides a direct view of how regulatory programs diverge even when the underlying gene repertoire is highly similar.

To summarize these structural differences, we computed a conservation score for each strain–iModulon pair, defined as the proportion of genes in that iModulon that are shared across all strains (Fig. 3a). While some iModulons have a broadly conserved composition, many display strain-specific differences in gene inclusion, indicating meaningful differences in TRN structure across these strains. In this section, we examine three representative iModulons, Flagella, Copper, and Anaerobic, that exhibit strong and clearly interpretable strain-specific differences in gene composition and activity, and connect to previously described phenotypic differences among the strains. All the iModulons can be found and further inspected in Table S3 or in our Github repository https://github.com/AnnieYuan21/Industrial-E.-coli-iModulons.

The first example is a flagella-associated iModulon identified in all six strains, representing a shared regulatory signal, yet its gene composition varied markedly (Fig. 3b). All iModulons contained most genes from the *flg* operon, encoding the hook and basal-body assembly machinery, while the inclusion of *fli*, *motAB*, and *che* genes responsible for additional basal-body, filament, motor, and chemotaxis components differed across strains (Morimoto and Minamino, 2014; Nakamura and Minamino, 2024). The reduced version of the flagella-associated iModulon in BL21 is consistent with prior reports that *E. coli* B derivatives carry insertion element-associated deletions encompassing the flagellar region, resulting in loss of motility (Studier et al., 2009) (red box in Fig. 3b). Strain C, however, carries the relevant genes in its genome but still lacks coordinated *fli*/*mot*/*che* membership, suggesting regulatory differences rather than gene absence.

Interestingly, the W3110 Flagella iModulon uniquely includes genes involved in LPS core and O-antigen biosynthesis (Fig. 3b, gene names highlighted in red). Defects in LPS core synthesis (*waaC*, *waaF*, or *waaG*) are known to abolish flagellar assembly in this strain, suggesting a regulatory linkage between envelope biogenesis and flagellar machinery (Wang et al., 2016). The presence of *waa*, *wbb*, and *rfb* genes in the flagella iModulon of W3110 points to a potential strain-specific coupling of motility and cell-surface remodeling that is not observed in the other strains. These compositional differences demonstrate how TRN structure could diverge through both genomic and regulatory variation across closely related strains.

A second example of strain-specific iModulon organization is the Copper iModulon. Copper homeostasis in *E. coli* involves conserved systems such as the CueR regulon and the Cus efflux machinery (Hyre et al., n.d.; Rensing and Grass, 2003). Some strains also carry additional resistance genes, *pco* and *sil*, typically on plasmids. In our dataset, only strains C and Crooks possess these genes, and in both cases, they are located on chromosomally integrated copper/silver tolerance islands (Blanco Massani et al., 2018; Brown et al., 1995; Król et al., 2019; Tetaz and Luke, 1983).

All six strains shared a core Copper iModulon, consisting primarily of the CusCBA efflux pump and CusF chaperone, and were activated upon CuSO_4_ exposure (Fig. 3c). In Crooks, the iModulon uniquely included the *pco* and *sil* genes, forming an expanded resistance set not observed in other strains (Fig. 3c, genes highlighted with red box). Notably, strain C carries the same *pco*/*sil* genes in its genome but neither expresses them under copper stress, nor includes them in its Copper iModulon.This could be indicative of differences in regulatory wiring, copper-sensing, or promoter activity (Blanco Massani et al., 2018; Bleichert et al., 2020). This contrast highlights that shared genomic content does not necessarily yield similar transcriptional responses. Although prior studies suggest that *pco*/*sil* genes do not substantially increase copper resistance under standard conditions (Chalmers et al., 2018; Santo et al., 2008), their transcriptional integration in Crooks reflects strain-specific regulatory activity that may become relevant in certain contexts.

Moreover, Crooks was the only strain whose Copper iModulon activity increased upon tryptophan supplementation (Fig. 3d). Among the amino acid supplementation conditions examined, tryptophan supplementation was the only one that elicited a notable increase in Copper iModulon activity in this strain. We hypothesize that this activation potentially suggests a relationship between tryptophan supplementation and copper detoxification capacity in this strain. A similar effect was previously reported in *E. coli* ATCC 25922, which lacks *pco* or *sil* (Bayne et al., 2024). This indicates that the tryptophan-linked improvement of copper tolerance is unlikely to be mediated by *pco/sil* genes, although the underlying mechanism might still be strain-specific.

**Fig. 4:**
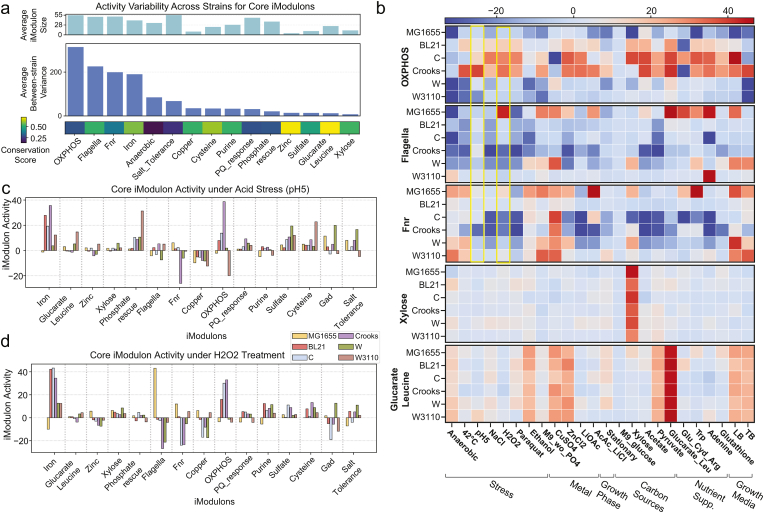
Differential iModulon Activation Patterns Across Strains. a) Average between-strain variance for all 15 core iModulons, capturing how strongly each iModulon's activity differs across strains under the same environmental condition. The top panel shows the size (gene number) of the corresponding iModulon, while the bottom panel shows the average conservation score (cross-strain average of the conservation scores defined in the section corresponding to Fig. 3a). **b)** Activity profiles of the three most variable core iModulons and the two least variable iModulons across all shared conditions in the dataset. Conditions include diverse stressors, carbon sources, and media supplements (annotated along the x-axis). The two conditions further examined in panels c and d are boxed in yellow. **c)** Activity levels for all core iModulons under the pH 5 condition, highlighting strain-dependent differences in acid stress responses. **d)** Activity levels for all core iModulons under the H_2_O_2_ condition, revealing how regulatory responses to oxidative stress vary across strains.

A third example is a prominent core iModulon that we termed the Anaerobic iModulon. It is conserved across all six strains but shows notable strain-to-strain variation in iModulon gene membership (Fig. 4e). This iModulon is enriched in GadX-regulated genes, such as *gadABC/E* and *hdeAB*, which contribute to acid resistance (Salgado et al., 2023; Sayed et al., 2007). In strain W, it is strongly activated under acidic conditions (pH 5). However, across all strains, its most pronounced activation occurs during anaerobic growth and stationary phase (Fig. S5), a pattern consistent with reports that *E. coli* upregulates anaerobic respiration and acid resistance mechanisms upon entry into stationary phase (Bouillet et al., 2024; Castanie-Cornet et al., 1999; Foster, 2004).

Differences in gene composition within this iModulon may help explain distinct anaerobic phenotypes among the strains. For example, strains C, Crooks, and W include multiple *hyc* genes encoding components of the formate hydrogen lyase (FHL) complex (gene names highlighted in red). FHL supports formate detoxification, redox balancing, and energy conservation under anaerobic, mildly acidic conditions (Bagramyan and Trchounian, 2003; McDowall et al., 2014; Peters and Sargent, 2023; Steinhilper et al., 2022). Interestingly, these same strains not only exhibit the highest anaerobic growth rates but also secrete the most formate (Monk et al., 2016). One potential hypothesis is that a rapid buildup of formate together with a decrease in external pH may activate genes in this iModulon. In turn, enriched FHL expression could potentially enable these strains to more effectively reprocess formate. In this context, such regulation may help prevent growth-limiting formate accumulation, support internal redox balance through hydrogen gas production, and possibly confer acid resistance by neutralizing excess protons during fermentation.

Furthermore, in BL21, the only strain capable of secreting lactate anaerobically, the Anaerobic iModulon uniquely includes *ldhA* (gene name highlighted in red), which encodes fermentative lactate dehydrogenase, further supporting the notion that iModulon structure can capture strain-specific fermentative strategies (Bunch et al., 1997; Monk et al., 2016). These observations show that strain-specific membership differences in the Anaerobic iModulon could potentially provide transcriptional evidence for distinct regulatory strategies governing anaerobic metabolism and acid stress response, offering a compelling transcriptome-level hypothesis for the observed differences in anaerobic phenotypes among closely related *E. coli* strains.

With these three examples, we demonstrate that core iModulons serving comparable biological functions are not identically constructed across strains. Instead, each strain assembles its own regulatory structure by grouping shared and strain-specific genes into different co-regulated sets, revealing clear TRN-structure differences that are consistent with their known physiological behaviors. This structural diversity underscores that regulatory organization varies among strains, even when gene repertoires are highly similar. The **M** and **A** matrices are included in Tables S1 and S2, enabling readers to explore particular cellular functions or carry out similar analyses tailored to their own research questions.

### 2.4 Core iModulon activity captures differential engagement of regulatory programs across strains

In addition to strain differences in iModulon composition described above, we observed substantial diversity in iModulon activities across the same conditions, capturing how cellular functions (represented by the iModulons) respond differently to environmental cues. A PCA of all iModulon activities across strains and conditions (Fig. S6) showed that each strain occupies a distinct region of the “activity space”, indicating how the strains differ in their global transcriptional responses. Whereas composition differences indicate changes in the underlying regulatory structure, activity differences capture how these iModulons are engaged in response to environmental cues. To quantify these differences, we ranked all iModulons by the variance of their activities across strains and matched conditions (Fig. 4a, middle panel). Several iModulons with well-characterized energy metabolism and stress-response roles displayed the highest strain-to-strain variability, including the OXPHOS, Flagella, and Fnr iModulons, followed by the Iron and Anaerobic iModulons. In contrast, the Xylose and Glucarate_Leucine iModulons showed low variance in their activation across strains. These intricate differences were also visible in the activation heatmaps across shared conditions (Fig. 4b).

Since TRN structural properties could contribute to these differences, we examined the average iModulon size and average conservation score (defined in Fig. 3a) alongside the variance ranking (Fig. 4a, top and bottom panels). Although some high-variance iModulons were larger or less conserved, these tendencies were not consistent across the dataset. This lack of consistency may reflect greater flexibility in larger or strain-specific iModulons, but neither property explained the variance ranking on its own. High- and low-variance iModulons occurred across the full range of iModulon sizes and conservation scores, indicating that divergent activation is not reducible to structural properties alone.

To further investigate these iModulon activity differences, we present the activity of all the core iModulons across strains under two stress conditions: acid stress (pH 5) and oxidative stress induced by H_2_O_2_ (Fig. 4c and d). Under pH 5, we observed pronounced variation in iModulon activation across strains. The Iron iModulon was activated in all strains except MG1655. This agrees with prior reports showing that MG1655 alters iron-related gene expression only under more severe acid stress (pH 4.4) and supports growing evidence that iron metabolism varies substantially across *E. coli* strains (Gao et al., 2023; Schumacher et al., 2023; Zhao et al., 2022). The Fnr iModulon, which contains genes involved in nitrate and nitrite respiration, showed strong repression in Crooks. This pattern suggests a possible strain-specific interaction between acid stress and the Fnr regulatory program in this background. In contrast, the OXPHOS iModulon was strongly activated in Crooks but strongly repressed in W3110, highlighting markedly different respiratory and energy-related transcriptional responses across strains (Fig. 4c). Although most industrial strains have not been transcriptionally profiled under acid stress, these divergent activation patterns suggest that different *E. coli* strains deploy distinct regulatory strategies under acidic conditions, balancing oxidative metabolism, anaerobic circuitry, and redox-linked processes.

Under H_2_O_2_-induced oxidative stress, MG1655 showed strong repression of the Iron iModulon, consistent with the established Fur-mediated repression of iron uptake and utilization genes through the *oxyRS* and *soxRS* systems (Matveeva et al., 2025; Zheng et al., 1999). In contrast, all other five industrial strains displayed strong activation of the Iron iModulon under the same treatment (Fig. 4d). To our knowledge, such activation has not been reported, indicating that these strains may regulate iron-associated functions differently from MG1655. Flagellar regulation also diverged substantially across strains. Several strains, especially Crooks and W, strongly repressed the Flagella iModulon, consistent with the notion that maintaining flagellar machinery imposes substantial energetic demands that cells often reduce during stress (Mei et al., 2017; Rodríguez-Rojas et al., 2020). MG1655, however, showed strong activation of the Flagella iModulon. Although previous studies have reported flagellar repression in MG1655 under high H_2_O_2_ concentrations, the 2 mM treatment used here may elicit a milder oxidative stress response in this strain (Abdelwahed et al., 2022). The cells might be engaging motility to escape from harmful environments (Rodríguez-Rojas et al., 2020). As with acid stress, the respiration-associated Fnr and OXPHOS iModulons showed markedly different responses across strains. Overall, oxidative stress reveals substantial divergence in the activation of major regulatory programs among the strains.

Together, these analyses of iModulon activation states demonstrate that the six industrial strains differ not only in the gene composition of their regulatory programs but also in how these iModulons are transcriptionally deployed across conditions. Some activation patterns match documented behaviors in the literature. Others exhibit distinct responses, underscoring that widely cited regulatory phenotypes in *E. coli* do not necessarily generalize across closely related strains. For many of the industrial strains, transcriptional responses to key stresses have not been reported previously, and the present analysis provides the first systems-view of how their regulatory programs are engaged. Full activity profiles for all iModulons across all strains and conditions can be found in Table S2.

### 2.5 Unique iModulons reflect strain-specific regulatory signals

In addition to the 15 core iModulons shared across all six strains, several strains contained a small number of unique iModulons, representing regulatory signals detected in only a single strain background. Across the dataset, BL21 exhibited three unique iModulons, while strains C, W, and W3110 each contributed one; MG1655 and Crooks showed no strain-specific iModulons. The limited number of unique iModulons could stem from the similarity of the experimental conditions applied across strains. Since all strains were profiled under a highly similar set of environmental cues, strain-specific regulatory programs that respond to strain-specific traits (e.g. sucrose utilization in W (Arifin et al., 2014a; Mohamed et al., 2019)) may not have been activated under the conditions tested. It is therefore possible that additional unique iModulons remain to be discovered under more diverse or strain-tailored conditions.

Although many of the unique iModulons contain largely uncharacterized genes (y-genes) (Sajid et al., 2024), several show recognizable signatures. One of the BL21-specific iModulons was enriched in phage-associated genes (Jeong et al., 2015; Studier et al., 2009), and the unique iModulon in W consisted largely of genes encoded on its plasmids (Archer et al., 2011). The W3110-specific iModulon was enriched in fimbrial and some flagellar genes and was strongly repressed under adenine treatment. Although no direct connection between fimbrial regulation and adenine sensitivity has been reported, W3110 is known to exhibit pronounced adenine sensitivity (Mosteller and Goldstein, 1975), and the transcriptional patterns observed here suggest a potential regulatory response worthy of further investigation. Notably, W3110 also displayed strong activation of its core Flagella iModulon under adenine treatment. Full gene memberships and activity profiles for all unique iModulons can be found in Table S3 or in our GitHub repository https://github.com/AnnieYuan21/Industrial-E.-coli-iModulons.

## 3 Discussion

The comparative framework developed here highlights regulatory diversity as a meaningful and previously underappreciated variation among industrial *E. coli* strains. By generating a matched transcriptomic compendium and applying a Multi-View ICA framework, we provide a unified, systems-level view of how regulatory organization and regulatory activation vary across six widely used production strains. Although these strains share highly similar genomes, their TRNs differ both in how regulatory programs are organized and in how those programs are used across conditions. These findings underscore that phenotypic differences commonly observed in industrial settings arise not only from gene content but also from differences in how strains coordinate, prioritize, and deploy transcriptional responses. This TRN-level perspective provides an intermediary viewpoint between genomic and physiological comparisons. It thus helps delineate genotype-phenotype differences between production strains.

A key insight from this work is that core iModulons with the same cellular functions are constructed differently across strains. Differences in iModulon gene membership indicates that closely related *E. coli* strains group and coordinate shared genes in distinct ways, leading to different patterns of coordination among cellular processes such as motility, metal homeostasis, or anaerobic metabolism. This observation emphasizes that regulatory organization itself can be a source of diversity in industrial hosts, independent of gene presence or absence.

Equally informative is the observation that the strains activate the same cellular function differently when exposed to the same environmental challenges. The contrasting responses under acid stress and peroxide treatment show that the strains do not share a single canonical *E. coli* response but instead draw on distinct combinations of iModulons to meet similar demands. This demonstrates that regulatory engagement itself differs across strains and adds another meaningful dimension to their overall regulatory diversity.

In addition, the matched-condition RNAseq compendium and Multi-View ICA framework used here establish a generalizable approach for examining regulatory diversity across related microbial hosts. As strain libraries expand and engineered variants become more common, such comparative analyses will be increasingly useful for evaluating differences in regulatory tendencies, identifying chassis with compatible transcriptional behaviors. Expanding the range of conditions or incorporating additional datasets in future work may further reveal strain-specific regulatory signals that were not apparent under the conditions examined here, offering an even broader perspective on regulatory diversity.

Within this context, our analysis focuses on transcriptional regulatory organization inferred from RNA-seq data and therefore captures regulation at the transcript level. However, cellular behavior is shaped by multiple interacting layers of regulation, including metabolic state, post-transcriptional processes, and protein-level dynamics. These additional layers can influence transcriptional activity through feedback mechanisms that are not explicitly represented in this framework. As such, the iModulon structure and activity described here provide a transcriptome-level view of regulatory organization that complements other mechanistic descriptions of cellular regulation.

In addition, the modular structure and activity profiles identified here may support future gene regulatory network modeling efforts. Recent studies have used iModulons as functional modules for strain engineering and have combined iModulon analysis with metabolic modeling to interpret transcriptome–phenotype relationships (Choe et al., 2024; Dalldorf et al., 2024; Rychel et al., 2023; Shin et al., 2024). These findings suggest that iModulon membership and condition-dependent activity profiles may provide useful constraints or quantitative inputs for modeling regulatory behavior across strains.

While transcriptional changes can reflect shifts in cellular state, their translation to measurable phenotypic outcomes depends on context, including the extent to which gene expression changes propagate through downstream metabolic and physiological processes. As such, iModulon activity is most informative when transcriptional regulation is a primary driver of the response, whereas additional regulatory layers may decouple transcription from phenotype in other settings. Future work integrating transcriptional regulatory analysis with metabolic modeling or targeted experimental validation may further strengthen these connections.

Taken together, this study offers a coherent way to interpret strain-specific behaviors at the transcriptome level and how they are manifested in phenotypic functions. It also provides new context for more informed strain selection and design by revealing transcriptional regulatory strategies that may align better or worse with specific process conditions, stressors, or production demands. Comparison of iModulon composition and activity across strains enables identification of regulatory differences associated with specific functions and conditions, providing testable hypotheses that can guide evaluation and prioritization of strains for specific applications. As such, it lays the groundwork for a more integrated view of microbial strain diversity in which regulatory insight guides both understanding and use of industrial hosts.

## 4 Methods

### 4.1 Strain selection

The six strains analyzed in this study (BL21, C, Crooks, MG1655, W, and W3110) were selected based on their widespread use as laboratory and industrial *E. coli* hosts and the availability of extensive prior genomic and phenotypic characterization. Together, these strains capture commonly used K-12 derivatives as well as non-K-12 production strains, enabling comparison across host backgrounds frequently used in practice. Strain W belongs to a different phylogroup, which we include to provide phylogenetic diversity within an otherwise closely related set. As a result, the analysis focuses on systematic comparison across commonly used hosts rather than exhaustive coverage of *E. coli* diversity, and conclusions should be interpreted within this scope.

### 4.2 Comparative genomic and phenotypic analyses

Complete genome sequences for all strains were obtained from public repositories (Archer et al., 2011; Clark et al., 2016; GenBank accession U00096.3; Freddolino et al., n.d.; Hayashi et al., 2006; Jeong et al., 2015; Król et al., 2019). The phylogenetic tree was generated with snippy and Gubbins with default parameters (Croucher et al., 2015; Seemann, 2020). Average Nucleotide Identity (ANI) was computed using FastANI (Jain et al., 2018), and the resulting pairwise ANI matrix was hierarchically clustered to generate an ANI dendrogram. Pangenome construction was performed using CD-HIT (Fu et al., 2012) with a sequence identity threshold of 0.9. Gene clusters identified by CD-HIT were further classified based on their presence across strains into core (present in all strains), accessory (present in more than one but not all strains), and strain-specific (present in only one strain) gene sets, which were used to generate Fig. 1c. For Biolog phenotypic microarray data, area-under-curve (AUC) values were obtained for each well from the kinetic measurements. For each strain and run, AUC values were then normalized by subtracting the corresponding negative-control well to account for baseline signal. Principal component analysis (PCA) was performed on the normalized Biolog AUC values, where each row corresponds to a strain and each column corresponds to a substrate condition. Prior to PCA, the data were z-score normalized. PCA was implemented using scikit-learn in Python.

### 4.3 Phenotype microarray assays

Cell suspensions were prepared following the Biolog protocol (PM Procedures for *E. coli* and Other Gram-Negative Bacteria). Single colonies were inoculated into M9 medium supplemented with 4 g/L glucose and grown overnight. Cells were washed twice with carbon-free M9 medium prior to use. For PM1 and PM2A plates, cell suspensions were adjusted to 42% transmittance (T) in carbon-free M9 medium containing 1X Dye A, then diluted to 85% T in the same medium. Aliquots (100 μL) were dispensed into each well. For PM3B and PM4A plates, the 85% T cell suspension was supplemented with 100X stocks of sodium succinate (final 20 mM) and ferric citrate (final 2 μM) prior to inoculation (100 μL per well).

### 4.4 Experimental condition selection for RNA-seq and data compendium compilation

The experimental conditions used in this study were selected to provide broad coverage of environmental, nutritional, and stress perturbations while maintaining a matched condition space across all six *E. coli* strains. Condition selection was guided primarily by PRECISE-1K, a large transcriptomic compendium of primarily *E. coli* MG1655 that includes diverse stresses, carbon and nitrogen sources, media formulations, and physiological states. From this repertoire, we selected representative conditions spanning major environmental and metabolic axes, including osmotic stress, acid stress, oxidative stress, nutrient limitation, and growth in alternative media, to ensure that the resulting dataset captured a wide range of transcriptional responses.

In addition to newly generated RNA-Seq profiles, we incorporated publicly available or previously generated datasets from our laboratory for each strain. These included transcriptomes from *fur* knockout strains (Gao et al., 2023), anaerobic growth samples (Monk et al., 2016), and evolved variants (all strains except strain C) (Kavvas et al., n.d.). All datasets were curated and integrated such that each strain was represented under a similar set of biological conditions even after quality control, allowing direct cross-strain comparison of transcriptional regulatory responses under matched conditions. The detailed conditions can be found in Table S4.

### 4.5 Media preparation and treatment details

All media were prepared according to the conditions listed in Table S4. For carbon-source experiments, individual carbon sources were added to M9 minimal medium lacking carbon. Additional supplements were incorporated into either M9 or LB medium as specified in Table S4. Media requiring low pH were adjusted to the indicated values using HCl. For stress treatment conditions, paraquat or NaCl stress was applied by adding the chemical directly to actively growing cultures when the optical density at 600 nm (OD_600_) reached 0.45. Cultures were incubated with the stressor for 30 min prior to sample collection.

### 4.6 Culture growth and sample collection

Overnight seed cultures were grown at 37 °C. The following day, fresh cultures were inoculated to an initial OD_600_ of 0.05 and grown under the specified conditions until they reached approximately OD_600_ ≈ 0.5. At OD_600_ ≈ 0.5, 3 mL of culture was immediately mixed with 6 mL of RNAprotect reagent. Cells were pelleted and stored at −80 °C until RNA extraction and RNA-Seq library preparation.

### 4.7 Total RNA preparation

Total RNA was prepared from cell pellets using a combination of reagents from Zymo Research and silica-coated magnetic beads from Luna Nanotech. First, 415 μL RNA Binding Buffer (Zymo Research) containing 8 μL Beta-mercaptoethanol was added to each 1.5 mL tube containing cell pellets. The pellets were dislodged into the buffer with an inoculating loop and then poured into 2 mL tubes containing around 250 μL 0.1 mm diameter zirconia/silica Beads (BioSpec Products). Any buffer remaining in the 1.5 mL tube after pouring was transferred by pipetting. Cells were lysed using an Omni International Bead Ruptor 12 instrument, 2 cycles, 1 min each at maximum speed of 6 m per second with a 1 min centrifugation at 10,000 x g between cycles. Lysates were then cleared by centrifugation for 2 min at 10,000 x g and 200 μL of the cleared lysates were transferred to tubes containing 400 μL 50% Ethanol and 20 μL silica-coated beads (Luna Nanotech). After briefly vortexing and incubating at room temperature for 2 min, the tubes were placed on a magnetic rack and the supernatants were aspirated once they were clear. The beads were washed once with RNA Wash Buffer (Zymo Research) and then each sample was resuspended in DNaseI master mix (Zymo Research DNA digestion buffer plus Zymo Research DNaseI). After a 15 min incubation at room temperature, 400 μL RNA Prep Buffer (Zymo Research) was added to each sample and vortexed briefly to mix. The tubes were then returned to the magnetic rack and then two washes with RNA Wash Buffer were followed by elution in 15 μL 10 mM Tris, pH 7.5. RNA quality and concentrations were checked with an Agilent TapeStation and Nanodrop respectively.

### 4.8 Ribosomal RNA removal

For each sample, 1 μg was used as input into the standard RiboRid procedure (Choe et al., 2021), which uses short, unmodified DNA oligonucleotide probes that selectively hybridize to ribosomal RNA, and Hybridase, a thermostable RNaseH enzyme (Biosearch Technologies), which degrades the ribosomal RNA. After stopping the reaction with the injection of RNA Binding Buffer into the samples as they are still at the high RNaseH reaction temperature, the samples were cleaned up with a 200 nt cutoff using an RNA Clean and Concentrator Kit (Zymo Research), substituting Luna Nanotech silica-coated magnetic beads for the columns. Ribosomal RNA-depleted RNA was eluted from the beads with 6 μL 10 mM Tris, pH 7.5.

### 4.9 RNA-seq library preparation and sequencing

A KAPA RNA HyperPrep Kit (Roche) was used to prepare RNAseq libraries from 5 μL of the ribosomal RNA-subtracted RNA following the manufacturer's protocol but using half volumes. The final PCR amplification step was modified by the addition of SYBR and the use of a qPCR instrument. Each sample was removed from the thermal cycler just before entering the plateau phase of amplification to avoid biases potentially introduced by reagent depletion. Library quality and concentrations were checked with an Agilent TapeStation and Qubit instrument respectively. Pooled libraries were run on an AVITI instrument (Element Biosciences).

### 4.10 Quality control and data normalization

Quality control procedures followed the established workflow described in the iModulonMiner framework (https://github.com/SBRG/iModulonMiner/tree/main/3_quality_control) (Sastry et al., 2024). Briefly, raw sequencing reads were evaluated for per-base sequence quality, per-sequence quality scores, per-base N content, and adapter contamination. Samples failing any of these criteria were removed from further analysis. After transcript quantification, we additionally assessed sample consistency by computing pairwise global correlations across all samples and replicate correlations within each condition. Samples with poor global agreement or replicate correlations below *r*^2^ < 0.9 were excluded.

Because our compendium integrated datasets generated across multiple sequencing batches and included public datasets from different projects, we performed normalization within each project. Each project included a designated reference condition, and log-TPM values were normalized relative to the corresponding project-specific reference to ensure comparability across strains and datasets.

After quality control, MG1655 has 69 samples with 35 unique conditions, BL21 has 75 samples with 35 unique conditions, C has 70 samples with 34 unique conditions, Crooks has 72 samples with 33 unique conditions, W has 76 samples with 31 unique conditions, and W3110 has 71 samples with 35 unique conditions. The number of conditions shared across all strains is 26 and can be found by comparing the metadata of the strains in Table S4.

### 4.11 Running multi-view ICA

Multi-View independent component analysis (Multi-View ICA) was performed using the MultiModulon package (version 0.2.0; https://github.com/Gaoyuan-Li/multimodulon).

For each strain, we loaded the normalized expression matrix (log_tpm_norm.csv; genes × samples), sample metadata, and reference-genome GFF annotation, and verified that the sample metadata matched the expression-matrix columns. Gene matches across strains were identified using bidirectional best hits (NCBI BLAST+) (Camacho et al., 2009). Gene annotations were loaded from GFF files, and all genes were re-annotated with eggNOG-mapper (v2.1.13, Cantalapiedra et al., 2021) to ensure consistency across genomes. We then applied a shared/individual multi-view ICA model based on the ShIndICA (Pandeva and Forré, 2023) framework to the aligned matrices. For each strain d, the aligned matrix X_d was centered and whitened separately to its numerical rank, and an orthogonal unmixing matrix W_d was estimated for that strain. Orthogonality constraints were enforced during optimization, and the objective combined a log-cosh non-Gaussianity term with a trace-based alignment term that encouraged the first c coordinates to be shared across strains. In each run, the first c coordinates were therefore treated as candidate shared/core components and the remaining a_d - c coordinates were treated as candidate strain-specific components for strain d. Optimization used full-batch L-BFGS with strong Wolfe line search (maximum 10,000 inner iterations), and component loading vectors were centered and variance-normalized after fitting.

Model dimensions were selected in two stages. First, the number of shared components c was optimized over a candidate grid from 5 to 65 in increments of 5 using 10 repeated ICA runs per candidate value. For each candidate k, we counted only components that passed a single-gene filter, defined as having the largest absolute gene weight no more than 3-fold greater than the second largest absolute gene weight. The final value of c was chosen from the elbow of this robust non-single-gene component curve. Second, with c fixed, the total number of components a_d for each strain was optimized separately by maximizing the number of robust strain-specific clusters recovered across repeated runs.

For the final decomposition, the model was run independently 10 times with different random seeds. After each run, components failing the single-gene filter were discarded, and retained components were sign-oriented so that the largest-magnitude loading was positive. Candidate core components from all strains and all runs were pooled and clustered with HDBSCAN (Campello et al., 2013) in Euclidean space. A robust core iModulon was retained only if the corresponding cluster showed sufficient support across runs and yielded a centroid for every strain. Candidate strain-specific components were clustered separately within each strain, and accepted cluster centroids defined the robust strain-specific iModulons for that strain. In this framework, core iModulons are defined by reproducible recovery of the shared latent component across all strains, whereas strain-specific iModulons are defined by reproducible recovery of components from the view-specific coordinates in only one strain. Gene membership within each retained iModulon was defined from the absolute M-matrix weights using quantile-filtered Otsu thresholding. Specifically, absolute gene weights were prefiltered at the 95th percentile, Otsu thresholding was then applied to the remaining weights. This step produced a component-specific gene-membership threshold and a binary presence matrix for downstream interpretation and cross-strain comparison. Importantly, a core iModulon in our analysis is defined by reproducible latent-component recovery across strains, not by requiring identical thresholded gene membership in every strain.

iModulons were named based on functional annotation of their gene composition, including enrichment of known pathways, regulons, or gene sets associated with specific biological processes. In cases where no clear functional enrichment was observed, iModulons were labeled as uncharacterized.

## CRediT authorship contribution statement

**Yuan Yuan:** Conceptualization, Data curation, Formal analysis, Investigation, Methodology, Project administration, Software, Visualization, Writing – original draft, Writing – review & editing. **Gaoyuan Li:** Data curation, Methodology, Software, Writing – review & editing. **Ying Hefner:** Data curation, Writing – review & editing. **Richard Szubin:** Data curation, Writing – review & editing. **Jaemin Sung:** Data curation, Writing – review & editing. **Irina Rodionova:** Data curation, Writing – review & editing. **Bernhard O. Palsson:** Conceptualization, Funding acquisition, Resources, Supervision, Writing – review & editing.

## Declaration of competing interest

The authors declare that they have no known competing financial interests or personal relationships that could have appeared to influence the work reported in this paper.

## Data Availability

All data and code used in this study are publicly available. RNA-seq datasets, processed data and analysis scripts are provided at https://github.com/AnnieYuan21/Industrial-E.-coli-iModulons, and details on running Multi-View ICA can be found at https://github.com/Gaoyuan-Li/multimodulon.
