# Supplementary material for: Transcriptional regulatory network differences between six industrial Escherichia coli strains
Source: Metab Eng Commun. 2026 Jun 9;23:e00283. doi: 10.1016/j.mec.2026.e00283 (PMC13316221; doi:10.1016/j.mec.2026.e00283)
Supplement: Multimedia component 1 [file mmc1.pdf]

Supplementary Information

Supplementary Figures

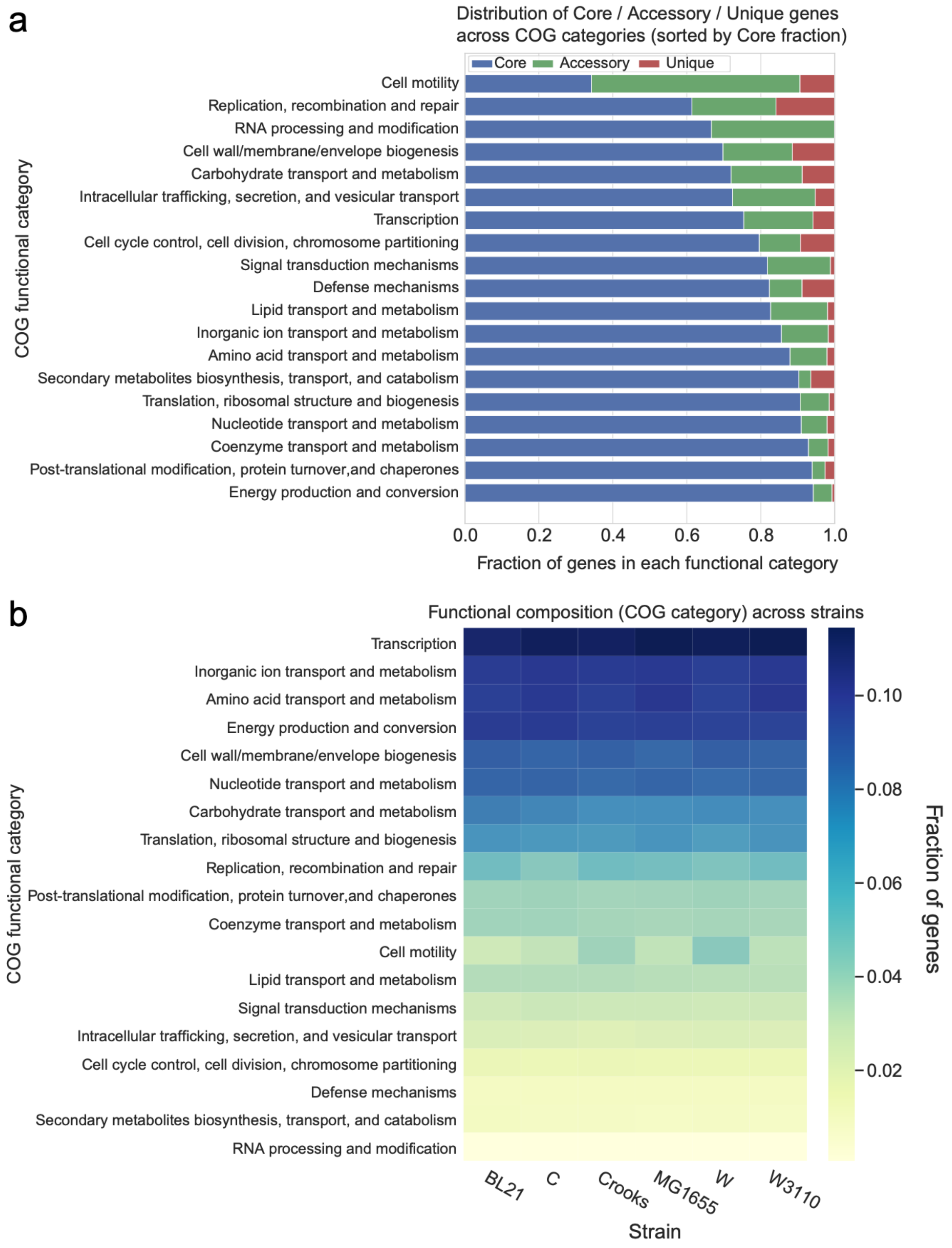

Fig. S1. Functional distribution of core, accessory, and unique genes and functional composition across strains. a). Distribution of core, accessory, and strain-specific genes across COG functional categories. Bars represent the fraction of genes in each category belonging to the core, accessory, or unique genome, with categories sorted from lowest to highest core fraction. **b).** Functional composition of each strain's genome across COG categories. The heatmap shows the fraction of genes assigned to each category for each strain.

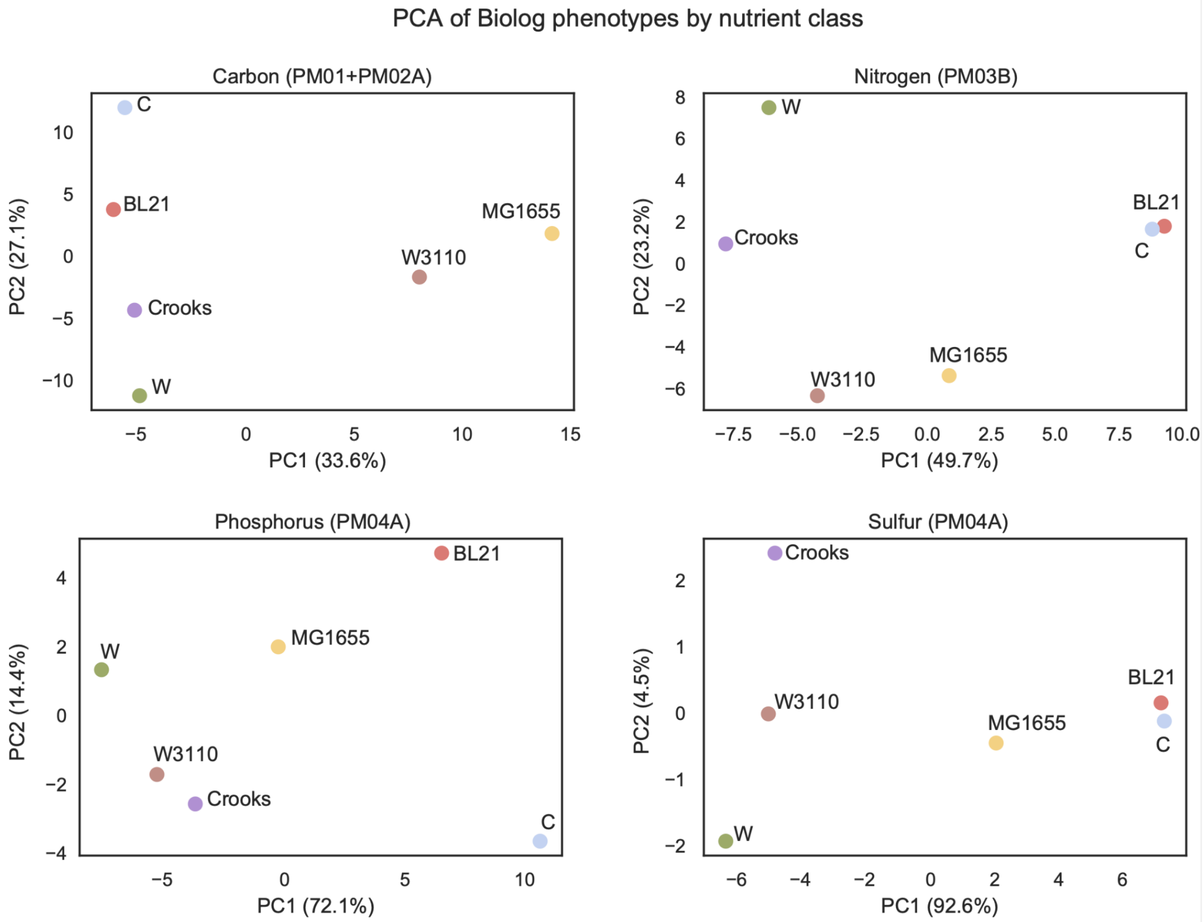

Fig. S2. PCA plot of Biolog utilization profiles for different substrate types. It can be noted that BL21 and C share similar utilization profiles for nitrogen and sulfur sources.

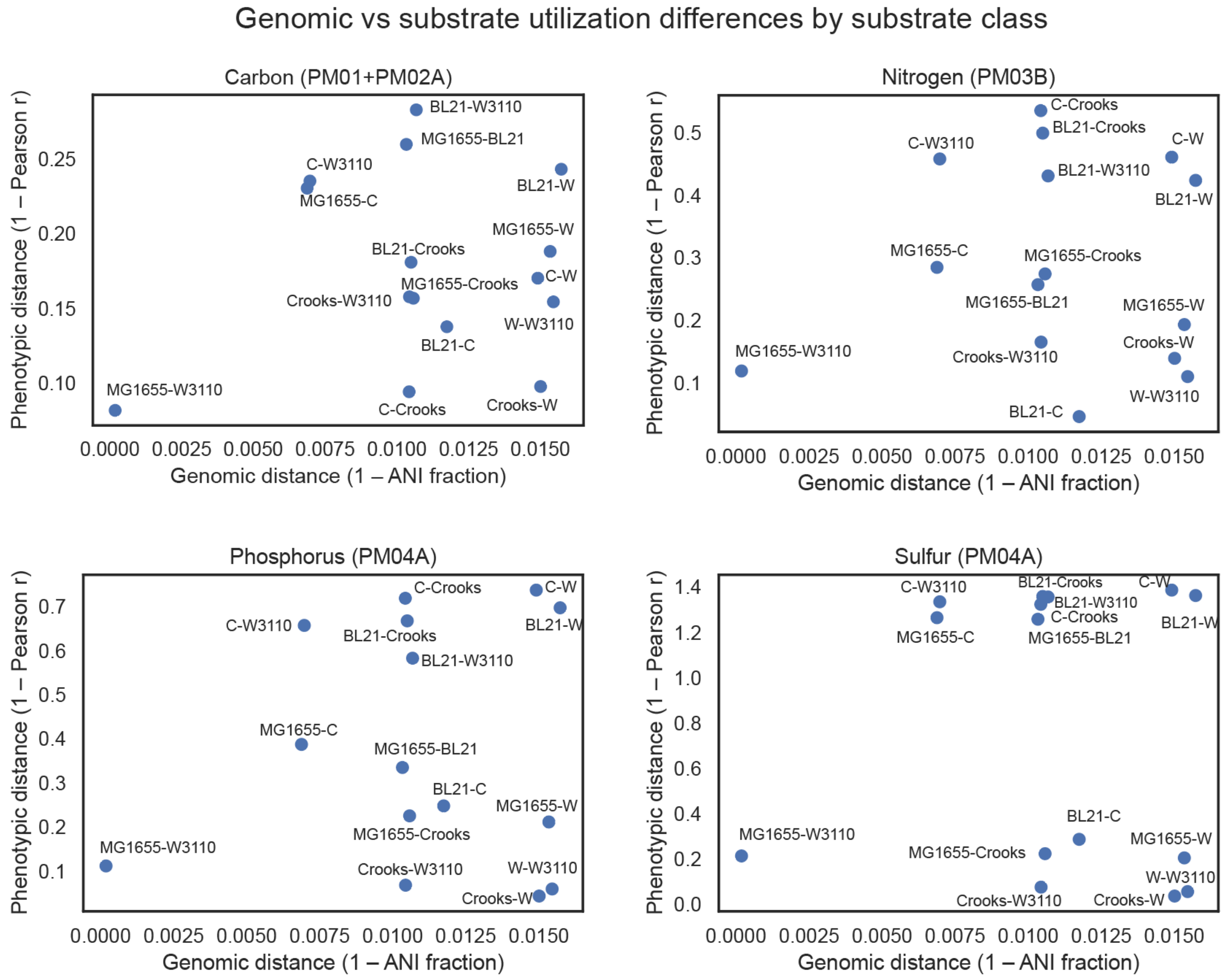

Fig. S3. Comparison of genomic distance and Biolog-based utilization difference for different substrate types

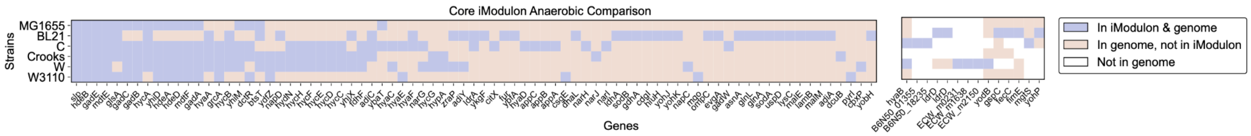

Fig. S4. Comparison of the core Anaerobic iModulon membership across six strains.

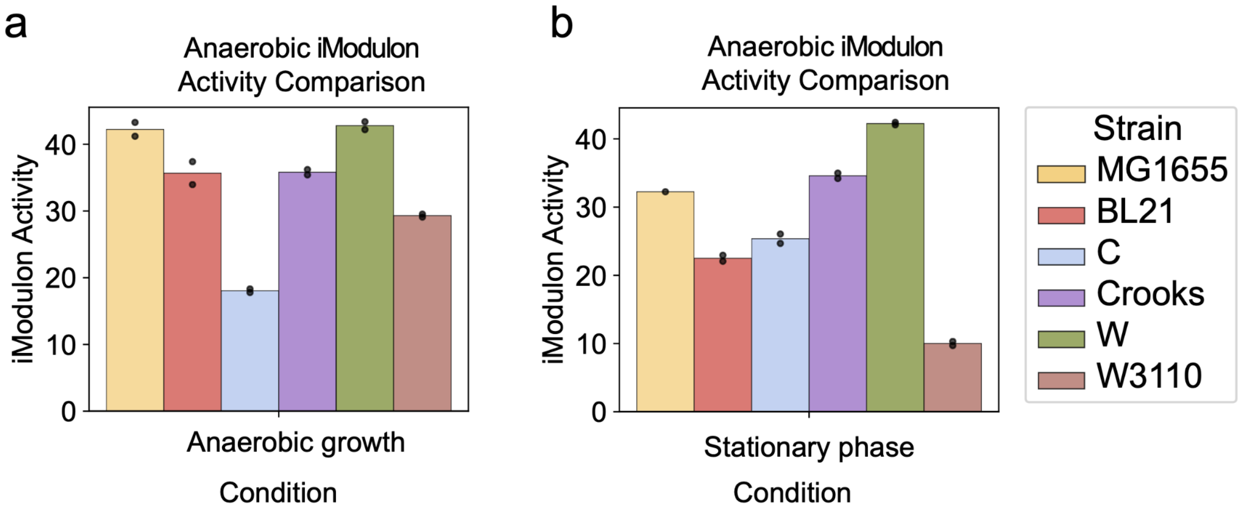

Fig. S5. Comparison of the core Anaerobic iModulon activity across six strains under a). anaerobic growth and b). stationary phase.

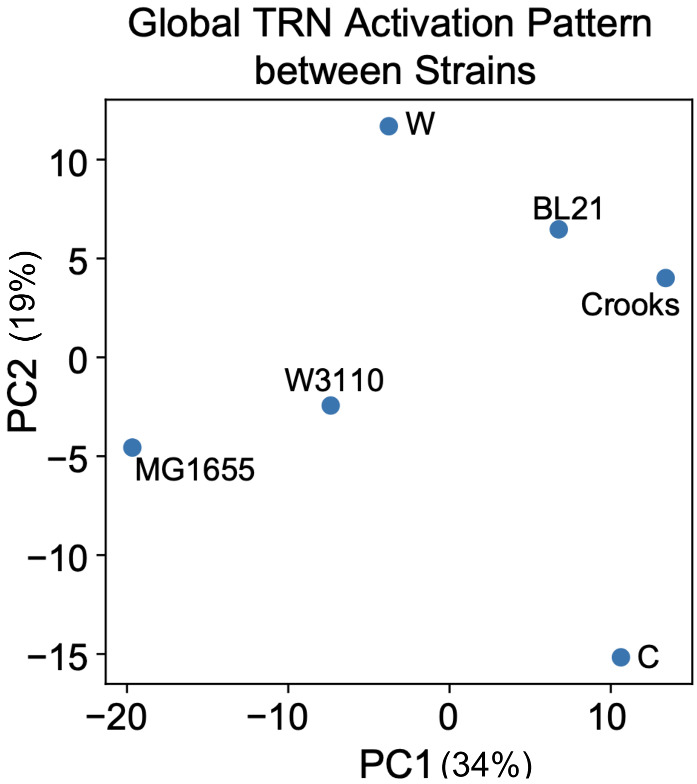

Fig. S6. A PCA of all iModulon activities across strains and conditions

Supplementary Tables

Table. S1. The M matrices for all six strains. The file contains six data sheets for the M matrices of all six strains.

Table. S2. The A matrices for all six strains. The file contains six data sheets for the A matrices of all six strains.

Table. S3. iModulon gene membership. The file contains six data sheets for all six strains. For each datasheet, the rows represent all genes in all iModulons, and the columns provide the common name of the gene, its gene product, and whether it is present or absent in a certain iModulon in that strain.

Table. S4. Detailed experimental conditions and metadata for all six strains. The sheet "Condition_Details" contains the detailed experimental conditions for all newly generated RNA-Seq samples. The rest of the data sheets outline the conditions included in each strain's dataset after quality control.
